# Psychotherapy and Placebos: Manifesto for Conceptual Clarity

**DOI:** 10.3389/fpsyt.2018.00379

**Published:** 2018-08-20

**Authors:** Charlotte R. Blease

**Affiliations:** ^1^Program in Placebo Studies, Beth Israel Deaconess Medical Center, Harvard Medical School, Boston, MA, United States; ^2^School of Psychology, University College Dublin, Dublin, Ireland

**Keywords:** psychotherapy, placebo, placebo effect, common factors across psychotherapies, philosophical psychology, thomas kuhn, placebo studies, placebo research

For nearly as long as the term has existed “placebo” has been a source of debate and disagreement. Scientists and philosophers have been active contributors to the protracted dialog about how best to define the term leading one prominent health researcher to argue that there appears to be “currently no widely accepted definition of placebo” (1). Meanwhile, new theoretical models aimed at resolving conceptual quagmires—once and for all [e.g., (1, 2)]—often seem to confound rather than crack the problem by inviting further questions (3, 4). If discussion about how to conceive placebo terminology seems to “rage” within the sphere of biomedicine (1) when it comes to the domain of psychotherapy conceptual entanglements appear even more complicated. Here the to-and-fro of debate has spanned the decades though been episodic rather than ongoing [e.g., (5–7)] and lately the debate has re-emerged [see: (8–11)]. Reviewing the recent contributions to this discussion, I argue that are indeed stable definitions for the terms *placebo* and *placebo effect* within the science of placebo studies. Furthermore, I argue that it is justified to use these definitions as a starting point for appraising conceptual disagreement, including the (apparently) contentious translation of these terms to psychotherapy. Exploring two provocative yet divergent claims about the relationship between placebo and psychological treatments (9, 10) I conclude that disagreement arises when researchers employ definitions of placebo that are disengaged from implicit scientific usage.

Discussion about conceptual or definitional matters in science may appear to be esoterica, however definitions are important. How we understand placebo concepts carries subtle but significant methodological significant methodological implications for clinical trials as well as for ethical practice in the delivery of care (4, 12, 13). Therefore, gaining clarity about the argumentation within disputes over concepts is not trivial—rather, it might even be viewed as a major priority for the field of placebo research.

In this *Mini Review* I focus on two of the most prominent recent claims about the relationship between placebo concepts and psychotherapy proposed by leading scholars (9–11). I argue that appearances to the contrary, the resultant conceptual quagmire is avoidable, and suggest how and why definitions of placebo and placebo effects have become muddled within the context of psychotherapy. However, to highlight why disagreement arises it is imperative to identify unambiguous definitions for the terms “placebo” and “placebo effect.” Fortunately, in this regard, the insights of philosopher of science Thomas Kuhn are instructive (14).

## Kuhn's insights

In the mid-Twentieth Century Thomas Kuhn helped to re-orientate philosophy of science by arguing that philosophers should move away from *a priori* formulations about the nature of science and pay closer attention to how scientists in fact reason and evaluate theories (15). One of Kuhn's most important (and least controversial legacies) is his claim that for empirical progress to arise in a given field of enquiry there must be discernible underlying conceptual stability (15). Following Kuhn and other post-positivist philosophers I argue that the most effective way to clarify conceptual issues is not to start from the philosophical armchair but to ask: How do established scientists *in fact* use these terms? (14).

If we assume that the field of placebo studies has emerged as an established field of science it is a small step to infer—despite the buzz of debate—that key definitions must be relatively settled in order to support the systematic growth of empirical knowledge. In a recent paper I argued that supposedly contested terminology is relatively settled in scientific placebo research (14).

However, before we address the question about what scientific usage reveals about core definitions, it is important to foreground the discussion by flagging up two important points. To begin, scientific concepts of placebo and placebo effect should be differentiated from those meanings ascribed to the terms in other non-expert domains including among medical practitioners, clinical investigators, patients, and research participants. Here I assume that even individuals who use placebos (e.g., in clinical trials, or as prescribing physicians) may not be experts in about how best to define these terms. Indeed, sociological research has demonstrated that both physicians and patients interpret “placebo” and “placebo effect” in myriad ways [see: (16, 17)]. How these non-experts define this terminology is important but not the present concern of this paper which is to inquire how these terms *ought* to be used.

Next, is a residual and paradoxical question: if there *is* conceptual consensus within the scientific placebo community about placebos and placebo effects why, then, is there so much debate about how to define the terms? I suggest three reasons. First, and perhaps most importantly the term “placebo” has been in use for centuries and is embedded within common lay as well as medical usage, further obstructing the possibility of clear and unambiguous meaning change. Second, even if we agree that scientific research into placebo effects is burgeoning—as a field of enquiry it has emerged only recently (14, 18). Therefore, we might still expect to observe a residual hangover of conceptual disputes regardless of whether such disagreement is substantive. Third, and related, even within scientific contexts, conceptual stability can be typified by implicit understanding rather than articulable, explicit definitions among many scientists who, as Kuhn observed, may be “little better than laymen at characterizing the established bases of their field, its legitimate problems and methods” [(15), p. 44].

Despite these challenges, I argue that we can discern conceptual stability over key definitions within the emergent science of placebo studies (14). I suggest that within the empirical research field, “*placebo effects* are understood to be positive health changes that occur as a result of specific psychobiological mechanisms …These psychobiological mechanisms are elicited, in turn, by a range of cues in the context of the practitioner-patient encounter” (14). Placebo effects, therefore, can be broadly understood as a natural kind of psychobiological phenomenon.

The term placebo is more nuanced. When it comes to placebo-randomized controlled trials (RCTs) the placebo allocation should ideally be identical to the verum intervention (the treatment under evaluation) in all respects except for its hypothesized remedial factor(s), and patient allocations should be randomized and double-blinded. The function of placebos in RCTs is to act as controls for the experimental “noise” that arises within clinical trials: this includes: regression to the mean, natural progression of an illness, patient or physician/investigator reporting biases, Hawthorne Effects, *as well as* placebo effects. Placebos in RCTs should therefore be conceived as a moving target: an instrument that is designed to mimic the appearance of a verum intervention (14). This means that the appearance and administration of the placebo control should always be dependent on the treatment under scrutiny rather than simply being reified as a particular kind of thing (e.g., “placebos are sugar pills”). Indeed, it would be less misleading to label placebos in RCTs as “control interventions” (4, 14). Placebo researchers also differentiate between *placebo effects* and *placebo responses* (12): the latter comprise the aggregate responses of receiving a placebo in an RCT—the factors associated with so-called “experimental noise” which, as noted, may or may not include placebo effects.

When it comes to the scientific community's definitions of placebos in clinical contexts, things get trickier. However, one place to glean insight is so-called *open-label placebo* experiments. Here the following script has been provided to patient participants in experimental set ups: “placebo pills are made of an inert substance, like sugar pills…have been shown in clinical studies to produce significant improvement in IBS symptoms through mind-body self-healing processes” [(19); see also: (20)]. In these scenarios, there are two implied definitions of placebo: (i) treatments theorized not to be effective for a condition or symptoms by virtue of their intrinsic properties; and secondly, the added notion that (ii) placebos—as described in (i)—*may be* causally implicated in the elicitation of placebo effects: here it is implied that placebos play a role as causal antecedents of psychobiological pathways which, when combined with other proximal conditions and factors in the context of health care (such as practitioner empathy, warmth, and confidence) cause placebo effect(s) (21). With these delineations in mind, I appraise two recently published, divergent analyses of the relationship between placebos and psychotherapy proposed by prominent scholars.

## “Aligning placebos and placebo effects with psychotherapy is incoherent”

The first claim owed to Kirsch, Wampold and Kelley is that deployment of this terminology within psychotherapy leads to a form of *reductio ad absurdum* (10). The authors argue: “In the context of medical treatment, placebo effects are relatively easy to define. They are the effects produced by factors other than the physical properties of the treatment” [(10), p. 123]. However, in psychological contexts, the authors contend, “Here is the central problem: The effect of psychotherapy is—by definition of the term *psychotherapy*—produced by something other than the physical properties of the treatment. Therefore, if we adhere to the received implicit definition of *placebo* as it has been used in the context of medicine, the effects of psychotherapy are ispo facto placebo effects and psychotherapy is ipso facto a placebo” [(10), p. 123].

First, Kirsch, Wampold and Kelley claim that we can rely on, “the received implicit definition of how placebo has been used in the context of medicine” [(10), p. 123]. Yet, as argued, implicit and explicit conceptualizations of placebos among *non-experts* are unhelpful precisely because the term has been deployed in myriad inconsistent and sometimes confusing ways [e.g., (17)]. To draw on another example, consider “folk biology” which encompasses among other intuitions, in-built ideas about how to classify species (22): this intuitive classification scheme does not provide a foolproof scientific foundation for how species are (in fact) related to one another. Mixing both classification systems would undermine scientific enquiry. Rather, to avoid conceptual quagmires, definitions of placebo must be anchored to how these terms are standardly, even if implicitly, deployed by experts working in the scientific placebo research community.

Second, the authors suggest that *all non-physical responses* to treatments should be conceived as placebo effects. This is incorrect: just because responses are non-physical—i.e., occurring at a *psychological* level—does not mean they are *de facto* placebo effects. This line of reasoning implies that every non-physical effect of a treatment is a placebo effect. Indeed, the logical extension of this argument is there can be no psychological responses *other than* placebo effects in psychotherapy: yet to suggest that the rich variety of psychological events elicited in psychotherapy simply amount to placebo effects is improbable (23). Correlatively, as scientific research in placebo studies has shown, not all *non-physical* responses to placebos are accurately described as *placebo effects*. We might surmise, in this instance, that Kirsch et al. confuse *placebo responses* with *placebo effects*.

Third, and finally, Kirsch, Wampold and Kelley argue, “[I]n evaluating the efficacy of psychotherapy, the placebo effect cannot and *should not be controlled*” [(10), p. 212]. From the premise that psychological responses *just are* equatable with placebo effects they infer the strong conclusion that it is *unjustified* to undertake placebo-RCTs of psychological treatments. This is unwarranted. From the definition of placebos as control interventions only the weaker claim is supported: *in principle* it is possible to design psychotherapy RCTs but *in practice*, the task is fraught with multiple serious challenges.

Indeed, one such problem is the double-blinding requirement (whereby neither therapist nor participant are aware about whether the individual has been allocated to placebo or the intervention under scrutiny). Another problem, which the authors highlight, is the need to control for so-called “common factors” in the delivery of psychotherapy. Here we must pause to consider what the term *common factors* means and how it should be distinguished from *specific treatment factors* in psychotherapy research.

Specific treatment factors vary according to different psychotherapy modalities and theories. So, for example, specific techniques in cognitive behavioral therapy (CBT) involve identifying hypothesized “cognitive distortions” or “maladaptive thoughts” which, according to CBT theorists and practitioners, are believed to have negative effects on behavior (24, 25). Here the goal of specific treatment techniques is to redress “faulty thinking” by promoting “cognitive restructuring” which, proponents of CBT theorize, will thereby elicit more psychologically constructive thoughts and behaviors (24, 25). Similarly, in psychodynamic psychotherapies and humanistic therapies distinctive kinds of specific techniques are theorized. *Common factors* on the other hand—and as the name suggests—refers to those features of treatment that appear to be shared across different psychological interventions. These include verbal and non-verbal therapist factors (e.g. empathy, positive regard); patient factors (e.g. confidence in the therapist); and factors associated with a strong working alliance between patient and psychotherapist.

To the extent that Kirsch et al. argue that controlling for common factors poses a serious obstacle to placebo-controlled clinical trials in psychotherapy we can agree with them. Nonetheless, conceivably this hindrance may yet be overcome. In the future, technological innovations may render it possible to delivery psychological treatments using avatars in the future: in such a scenario, we might hypothesize that the regulation and control of common factors would become practicable within psychotherapy-RCTs.

## “Psychotherapy is a placebo”

Gaab et al. (9) and Trachsel and Gaab (11) present a very different interpretation of the relationship between placebo and psychotherapy. Their proposition is that psychotherapy has an “unwanted proximity” to placebos which poses problems for ethical clinical practice in respect of disclosures to patients about how psychotherapy works [(11), p. 493]. Here I will focus on the claim that psychotherapy is interpretable as a “placebo” and sidestep intricate questions about ethical implications of this conjecture (26). Since these arguments rely on: (a) common factors research into psychotherapy; and (b) Grünbaum's model of placebos (6), it is first necessary to set the scene by providing an overview of each premise.

### Common factors research

Empirical findings indicate that different versions of psychotherapy, which employ different treatment techniques, appear to be equally successful (23). This is often referred to as the “Dodo Bird Verdict”—the label is derived from the words of the Dodo Bird in Alice in Wonderland: “everybody has won and all must have prizes” [(27), p. 995]. Subsequently, it has been proposed that the Dodo Bird Verdict is explained by the common factors hypothesis—namely, that it is the common factors and not the specific factors that are relevant to outcome (23). While the Dodo Bird Verdict is still somewhat contested (28) a considerable body of research nonetheless suggests that the common factors play a significant role in mediating treatment outcomes (29, 30).

### Grünbaum's model of placebo

The second key idea underpinning Gaab et al. (9) and Trachsel and Gaab's (11) views about the relationship between psychotherapy and placebos, is Adolph Grünbaum's model of placebos. Grünbaum differentiates between “characteristic” and “incidental” features of interventions which he says must be relativized to—that is, determined by—particular theories about how treatments work [(6), p. 33]. So, characteristic factors of (for example) *amoxicillin* are its particular antibiotic formula in the pill; meanwhile, the incidental factors include its coloration, the bulking agent, and price. Placebos, on this framework, are conceived as interventions that lack any remedial *characteristic* treatment factors for a particular condition. Placebo effects, on the other hand, are conceived as those positive effects that arise from the *incidental* features of a treatment.

Embracing the validity of Grünbaum's model and the common factors hypothesis, Gaab et al. argue that the specific techniques of psychotherapy can be equated with Grünbaum's description of characteristic features of treatments and the common factors interpreted as incidental factors (9, 11). From this perspective, it is concluded that psychotherapy risks being conceived as a placebo.

What should we make of this analysis? A positive feature of Grünbaum's framework is his conceptualization of placebos as a moving classification: placebos are not reified as physical “things” e.g., sugar pills. But when it comes to placebo effects problematic discrepancies arise between Grünbaum's model and scientific research. On Grünbaum's account placebo effects are also conceived as moving targets (rather than as a natural kind): this is because they are conceived as the effects of *incidental treatment factors* associated with a particular treatment theory. Even if we modify and narrow this framework to accommodate the view that placebo effects are the *positive effects of incidental factors* (1) the account is still too liberal from the perspective of scientific placebo studies. This is because other positive psychological effects may conceivably be precipitated by incidental factors (e.g., reporting biases or Hawthorne effects which precipitate positive health behaviors, Pygmalion effects, and/or other psychological processes).

Further problems arise when applying this model to psychotherapy research. If: (a) we accept the validity of the common factors hypothesis; and (b) defend Grünbaum's model of placebos, it might be countered that the common factors cannot be interpreted as “incidental factors.” This is because theories within psychotherapy typically regard common factors as integral components of treatment [e.g., (25)]. Thus, the terms *specific* and *common factors* may not be construed as conceptually isomorphic with Grünbaum's *characteristic* and *incidental factors*, respectively. Instead, it would be more accurate to describe different versions of therapy as employing *idiosyncratic* treatment factors alongside common factors but that all of these factors are “characteristic,” i.e., considered to be necessary factors for psychotherapy to be successful.

## Conclusions and recommendations

In conclusion, conceptual clarity in placebo studies will only be settled when we attend to how placebo terminology is *in fact* used within burgeoning scientific research—rather than how disputants say it used.

In examining the relationship between psychotherapy and placebos we must ask: What is the context of our analysis? Placebos in clinical trials, I have argued, are best characterized as control interventions. As with all control interventions, then, the function of placebos in psychotherapy clinical trials is to mimic the appearance of the verum treatment except for its particular, hypothesized, remedial factor. In practice, designing placebos for psychotherapy clinical trials is hugely challenging; though (as suggested) future technological innovations may eventually help to resolve recalcitrant problems.

In clinical contexts it is incorrect to describe psychotherapy as a placebo. Within the scientific placebo field, researchers implicitly define placebos as, “treatments theorized not to be effective for a condition or symptoms by virtue of their intrinsic properties.” While research into basic science of psychotherapy mechanisms is not advanced, it appears that common factors play an important role in mediating change. Moreover, these factors are also theorized by proponents of different psychological treatments to be necessary to outcome.

Finally, placebo effects cannot be equated with “all non-physical responses” of a treatment. The growing science of placebo studies informs us that placebo effect(s) are the remedial outcomes of specific psychobiological mechanisms. Such mechanisms may be elicited by psychotherapy—just as they may be triggered in other treatment modalities.

## Author contributions

The author confirms being the sole contributor of this work and approved it for publication.

### Conflict of interest statement

The author declares that the research was conducted in the absence of any commercial or financial relationships that could be construed as a potential conflict of interest.
